# Generalized *β*−Hyers–Ulam–Rassias Stability of Impulsive Difference Equations

**DOI:** 10.1155/2022/9462424

**Published:** 2022-09-15

**Authors:** Yahya Almalki, Gul Rahmat, Atta Ullah, Fatima Shehryar, Muhammad Numan, Muhammad Usman Ali

**Affiliations:** ^1^Department of Mathematics, College of Sciences, King Khalid University, Abha 61413, Saudi Arabia; ^2^Department of Mathematics, Islamia College Peshawar, Peshawar, Pakistan; ^3^Department of Mathematics, COMSATS University Islamabad, Attock Campus, Attock, Pakistan

## Abstract

This paper describes the existence and uniqueness of the solution, *β*-Hyers–Ulam–Rassias stability and generalized *β*-Hyers–Ulam–Rassias stability of an impulsive difference system on bounded and unbounded discrete intervals. At the end, an example is given to illustrate the theoretical result.

## 1. Introduction

Many physical problems can be expressed in mathematical models using differential equations. Differential equations enable us to study the rapid changes in physical problems, for example, blood flows, river flows, biological systems, control theory, and mechanical systems with impact. A system of differential equations with impulses can be used to model several above-listed problems. A few existing results for a general class of impulsive systems were discussed by Ahmad [1]. The theory of impulsive difference equations was studied in [2–4]. In [5], the existence of solutions for semilinear abstract differential equations without instantaneous impulses was discussed.

At the University of Wisconsin, Ulam [6] proposed the stability problem, stated as follows. Let us denote by *H*_1_ the group and by *H*_2_ the metric group with a metric *δ* and a constant *ν* > 0. The problem is to study if there exists *λ* > 0 satisfies for every *h* : *H*_1_⟶*H*_2_ such that

(1) $$\begin{array}{c} \delta \left(h\left(\sigma \nu \right),h\left(\sigma \right)h\left(\nu \right)\right)\leq \lambda ,\;\forall \sigma ,\;\nu \in H_{1}, \end{array}$$

there exists a homomorphism *f* : *H*_1_⟶*H*_2_ that satisfies

(2) $$\begin{array}{c} \delta \left(h\left(\sigma \right),f\left(\sigma \right)\right)\leq \nu ,\;\forall \sigma \in H_{1}. \end{array}$$

The linear functional equations, of the form *f*(*x*+*y*)=*f*(*x*)+*f*(*y*), and their solutions have been discussed in several spaces. A linear transformation is a solution of a linear functional equation. By considering the *H*_1_ and *H*_2_ as Banach spaces, Hyers [7] discussed the above problem in terms of linear functional equations. Then, Aoki [8] and Rassias [9] extended the concept of Hyers and Ulam. In the last decade, we have seen some worthwhile generalizations in the direction of Ulam stability.

In 2012, Wang et al. [10] studied the Ulam-type stability of first-order nonlinear impulsive differential equations by utilizing the bounded interval with finite impulses. In 2014, Wang et al. [11] studied the Hyers–Ulam–Rassias stability and generalized Hyers–Ulam–Rassias stability for impulsive evolution equations on a closed and bounded interval. In 2015, Zada et al. [12] studied the Hyers–Ulam stability of differential systems in terms of a dichotomy. The existence and Hyers–Ulam stability of the periodic fractional stochastic and Riemann–Liouville fractional neutral functional stochastic impulsive differential equations were given [13, 14]. Recently, Rahmat et al. [15] studied the Hyers–Ulam stability of delay differential equations. In 2019, Hu and Zhu [16] presented the stability criteria for an impulsive stochastic functional differential system with distributed delay-dependent impulsive effects. Furthermore, Hu et al. [17] provided the improved Razumikhin stability criteria for an impulsive stochastic delay differential system, and for a detail study, we refer to the readers to [17] and the references therein.

In this paper, we will explain the *β*-Hyers–Ulam–Rassias stability and generalized *β*-Hyers–Ulam–Rassias stability of the impulsive difference system of the form

(3) $$\begin{array}{c} \left\{\begin{array}{cc} \Theta_{n+1}=H\Theta_{n}+B\zeta_{n}+f\left(n,\Theta_{n},\zeta_{n}\right), & n\geq 0,\\ \Theta_{0}, & n=0,\\ \Theta_{n_{k}+1}=\Theta_{n_{k}-1}+I_{k}\left(n,\Theta_{n_{k}-1},\zeta_{n_{k}-1}\right), & k=1,2,3,\ldots ,m, \end{array}\right. \end{array}$$

where the constant matrix *H*, *B* ∈ *ℝ*^*n*×*n*^, *f* ∈ *ℂ*(*ℤ*_+×*X*,*X*_) and Θ_*n*_ ∈ *B*(*ℤ*_+_, *𝕏*) space of bounded and convergent sequences, *ℤ*_+_={0,1,2,…} and *𝕏*=*ℝ*^*n*^, *I*={0,1,2,…, *n*}. In fact, we are presenting a discrete version of the work given in [18], in which *β*-Hyers–Ulam–Rassias stability was discussed for differential equations. With the help of [15, 18], we find out *β*-Hyers–Ulam–Rassias stability of the difference equation.

## 2. Preliminaries

Here, we discuss some notation and definitions, which will be needed for our main work. The *n*-dimensional Euclidean space will be denoted by *ℝ*^*n*^ along with the vector norm ‖·‖, and *n* × *n* matrices with real-valued entries will be denoted by *ℝ*^*n*×*n*^. The vector infinite norm is defined as ‖*v*‖=max_1≤*i*≤*n*_|*v*_*i*_|, and the matrix infinite-norm is given as ‖*A*‖=max_1≤*i*≤*n*_∑_*j*=1_^*n*^|*a*_*ij*_| where *v* ∈ *ℝ*^*n*^ and *A* ∈ *ℝ*^*n*×*n*^, also *v*_*i*_ and *a*_*ij*_ are the elements of the vector *v* and the matrix *A*, respectively. *ℂ*(**I**, *𝕏*) will be the space of all convergent sequences from **I** to *𝕏* with norm ‖*v*‖=sup_*n*∈**I**_‖*v*_*n*_‖. We will use *ℝ*, *ℤ*, and *ℤ*_+_ for the set of all real, integer, and nonnegative integer numbers, respectively. The next lemma is a basic result about the solution of the difference system (1).

**Lemma 1 .**

The impulsive difference system (1) has the solution

(4) $$\begin{array}{c} X_{n}=H^{n}\Theta_{0}+H^{n-1}\sum_{i=0}^{n-1}H^{-i}B\zeta_{i}+H^{n-1}\sum_{i=0}^{n-1}H^{-i}f\left(i,\Theta_{i},\zeta_{i}\right)\\ +\sum_{n_{k}=0}^{n}T\left(n-n_{k}\right)I_{k}\left(n_{k},\Theta_{n_{k}},\zeta_{n_{k}}\right),\;n\in I. \end{array}$$

The solution can easily be obtained by consecutively placing the values of *n* ∈ {0,1,2,…}.

**Definition 1 .**

A function ‖·‖_*β*_ : *𝕍*⟶[0, *∞*) is called *β*-norm, with 0 < *β* ≤ 1, where *𝕍* is a vector space over the field **K**, if the function satisfied the following properties:

1. ‖ℋ‖_*β*_=0 if and only ℋ=0
2. ‖*κ*ℋ‖_*β*_=|*κ*|^*β*^‖ℋ‖_*β*_,  foreachk *κ* ∈ **K** and ℋ ∈ *𝕍*
3. ‖ℋ+ℋ_1_‖_*β*_ ≤ ‖ℋ‖_*β*_+‖ℋ_1_‖_*β*_,  forall ℋ,  ℋ ∈ *𝕍*

And (*𝕍*, ‖·‖_*β*_) is said to be *β*-norm space.

**Definition 2 .**

Let *ϵ* > 0, *ψ* > 0 and *φ*_*n*_ ∈ *B*(*I*, *X*). A sequence Θ_*n*_ will be an *ϵ*-approximate solution of (1), if

(5) $$\begin{array}{c} \left\{\begin{array}{cc} \left\|\Theta_{n+1}-H\Theta_{n}-B\zeta_{n}-f\left(n,\Theta_{n},\zeta_{n}\right)\right\|\leq \varepsilon \varphi_{n}, & n\geq 0,\\ \left\|\Theta_{n_{k}+1}-\Theta_{n_{k}-1}-I_{k}\left(n,\Theta_{n_{k}-1},\zeta_{n_{k}-1}\right)\right\|\leq \varepsilon \psi , & k=1,2,3,\ldots ,m, \end{array}\right.. \end{array}$$

**Definition 3 .**

System (1) is said to be *β*-Hyers–Ulam–Rassias stable if for every *ϵ*-approximate solution *Y*_*n*_ of system (1), there exists an exact solution Θ_**n**_ of (1) and a nonnegative real number *𝒞*_*f*,*N*,*M*,*φ*,Ψ_ such that

(6) $$\begin{array}{c} {\left\|Y_{n}-\Theta_{n}\right\|}^{\beta }\leq C_{f,N,M,\varphi ,\Psi }\varepsilon^{\beta }\left(\varphi_{n}^{\beta }+\psi^{\beta }\right),\mathrm{for}\;\mathrm{all}\;n\in I. \end{array}$$

**Definition 4 .**

System (1) is a generalized Hyers–Ulam–Rassias stable if for every *ϵ*-approximate solution *Y*_*n*_ of system (1), there will be an exact solution Θ_*n*_ of (1) and a nonnegative real scalar *𝒥*_*M*,*η*_Ψ_,*η*_*ϕ*_,*f*_ such that

(7) $$\begin{array}{c} {\left\|Y_{n}-\Theta_{n}\right\|}^{\beta }\leq J_{M,\eta_{\Psi },\eta_{\phi },f}\varepsilon^{\beta }\left(\varphi_{n}^{\beta }+\Psi_{k+1}^{\beta }\right),\;n\in I. \end{array}$$

**Remark 1 .**

From (2), it is clear that **Y** ∈ *ℂ*(**I**, **X**) satisfies (2) if and only if there exists h ∈ *ℂ*(**I**, **X**) and a sequence *h*_*k*_, *k* ∈ *M* satisfying

(8) $$\begin{array}{c} \left\{\begin{array}{c} \left\|h_{n}\right\|\leq \varepsilon \psi_{n},\;n\in M,\\ \Theta_{n+1}=H\Theta_{n}+B\zeta_{n}+f\left(n,\Theta_{n},\zeta_{n}\right)+h_{n},\;n\in {\mathbb{Z}}_{+},\\ Y_{0}=\Theta_{0}+h_{0},\\ \Theta_{n_{k}+1}-\Theta_{n_{k}-1}=I_{k}\left(\Theta_{n_{k}-1},\zeta_{n_{k}-1}\right)+h_{n_{k}},\;k\in M. \end{array}\right. \end{array}$$

The solution of Remark 1 is

(9) $$\begin{array}{c} \Theta_{n}=H^{n}\left(\Theta_{0}+h_{0}\right)+H^{n-1}\sum_{i=0}^{n-1}H^{-i}B\zeta_{i}+H^{n-1}\sum_{i=0}^{n-1}H^{-i}\left(f\left(i,\Theta_{i},\zeta_{i}\right)+h_{i}\right)\\ +\sum_{n_{k}=0}^{n}T\left(n-n_{k}\right)\left(I_{k}(n_{k},\Theta_{n_{k}},\zeta_{n_{k}}\right)+h_{n_{k}},\;n\in I. \end{array}$$

**Lemma 2 (see [19]).**

for any *n* ≥ 0 with

(10) $$\begin{array}{c} \left\|U_{n}\right\|\leq a_{n}+\sum_{i=0}^{n}P_{i}U_{i}+\sum_{0\leq n_{k}\leq n}\gamma_{k}U_{n_{k}-1},\;n\geq 0, \end{array}$$

then, we have

(11) $$\begin{array}{c} U_{n}\leq a_{n}{\left(1+\gamma_{k}\right)}^{k}\exp\left(\sum_{i=0}^{n}P_{i}\right),\;\mathrm{where}\;k\in I. \end{array}$$

**Remark 2 .**

If we replace *γ*_*k*_ by *γ*_*k*_*n*__, then

(12) $$\begin{array}{c} Y_{n}\leq c\prod_{n_{k}=0}^{n}\left(1+\gamma_{k_{n}}\right)\exp\left(\sum_{i=0}^{n}P_{i}\right)\;\mathrm{for}\;n\geq 0. \end{array}$$

## 3. Uniqueness and Existence of Solution of an Impulsive Difference System

To describe the uniqueness and existence of the solution of system (1), we will use the following assumptions:

*G*_1_: for *f*, *I*_*k*_ : *ℤ*_+×*X*×*X*_⟶*𝕏*, *k* ∈ *I*, there exist constants ℒ_*f*_ > 0 and ℒ_*I*_*k*__ > 0, such that
  (13) $$\begin{array}{c} \left\|f\left(n,ℑ,p\right)-f\left(n,{ℑ}^{\prime },p\right)\right\|\leq {ℒ}_{f}\left\|ℑ-{ℑ}^{\prime }\right\|,\\ \left\|I_{k}\left(n,w,y\right)-I_{k}\left(n,w^{\prime },y\right)\right\|\leq {ℒ}_{I_{k}}\left\|w-w^{\prime }\right\|. \end{array}$$
*G*_2_:
  (14) $$\begin{array}{c} M=\underset{i\in I}{\text{sup}}\left\|H^{i}\right\|,\;N=\underset{0\leq n_{k}\leq n}{\max}\left\|T\left(n-n_{k}\right)\right\|. \end{array}$$
*G*_2_^*∗*^:
  (15) $$\begin{array}{c} \left[M\sum_{i=0}^{n-1}\left\|H^{-i}\right\|{ℒ}_{f}+Nn{ℒ}_{I_{k}}\right]<1. \end{array}$$

**Theorem 1 .**

If assumptions *G*_1_, *G*_2_, and *G*_2_^*∗*^ are held, then system (1) has a unique solution Θ ∈ *ℂ*(**I**, *𝕏*).

**Proof**

Define *𝒜* : *ℂ*(**I**, *𝕏*)⟶*ℂ*(**I**, *𝕏*) by

(16) $$\begin{array}{c} A\Theta_{n}=H^{n}\Theta_{0}+H^{n-1}\sum_{i=0}^{n-1}H^{-i}B\zeta_{i}+H^{n-1}\sum_{i=0}^{n-1}H^{-i}f\left(i,\Theta_{i},\zeta_{i}\right)\\ +\sum_{n_{k}=0}^{n}T\left(n-n_{k}\right)I_{k}\left(n_{k},\Theta_{n_{k}-1},\zeta_{n_{k}-1}\right). \end{array}$$

Now, for Θ, Θ′ ∈ *ℂ*(**I**, *𝕏*), we have

(17) $$\begin{array}{c} \left\|A\Theta_{n}-A\Theta_{n}^{\prime }\right\|=\left\|\begin{array}{c} H^{n}\Theta_{0}+H^{n-1}\sum_{i=0}^{n-1}H^{-i}B\zeta_{i}+H^{n-1}\sum_{i=0}^{n-1}H^{-i}f\left(i,\Theta_{i},\zeta_{i}\right)\\ +\sum_{n_{k}=0}^{n}T\left(n-n_{k}\right)I_{k}\left(n_{k},\Theta_{n_{k}-1},\zeta_{n_{k}-1}\right)\\ -H^{n}\Theta_{0}-H^{n-1}\sum_{i=0}^{n-1}H^{-i}B\zeta_{i}-H^{n-1}\sum_{i=0}^{n-1}H^{-i}f\left(i,\Theta_{i}^{\prime },\zeta_{i}\right)\\ -\sum_{n_{k}=0}^{n}T\left(n-n_{k}\right)I_{k}\left(n_{k},\Theta_{n_{k}-1}^{\prime },\zeta_{n_{k}-1}\right) \end{array}\right\|. \end{array}$$

This implies that

(18) $$\begin{array}{c} \left\|A\Theta_{n}-A\Theta_{n}^{\prime }\right\|=\left\|H^{n-1}\sum_{i=0}^{n-1}H^{-i}f\left(i,\Theta_{i},\zeta_{i}\right)+\sum_{n_{k}=0}^{n}T\left(n-n_{k}\right)I_{k}\left(n_{k},\Theta_{n_{k}-1},\zeta_{n_{k}-1}\right)\right.\\ -\left.H^{n-1}\sum_{i=0}^{n-1}H^{-i}f\left(i,\Theta_{i}^{\prime },\zeta_{i}\right)-\sum_{n_{k}=0}^{n}T\left(n-n_{k}\right)I_{k}\left(n_{k},\Theta_{n_{k}-1}^{\prime },\zeta_{n_{k}-1}\right)\right\|\\ \leq +\sum_{n_{k}=0}^{n}\left\|T\left(n-n_{k}\right)\right\|\left\|I_{k}\left(n_{k},\Theta_{n_{k}-1},\zeta_{n_{k}-1}\right)-I_{k}\left(n_{k},\Theta_{n_{k}-1}^{\prime },\zeta_{n_{k}-1}\right)\right\|\\ \leq M\sum_{i=0}^{n-1}\left\|H^{-i}\right\|{ℒ}_{f}\left\|\Theta_{i}-\Theta_{i}^{\prime }\right\|+N\sum_{n_{k}=0}^{n}{ℒ}_{I_{k}}\left\|\Theta_{n_{k}-1}-\Theta_{n_{k}-1}^{\prime }\right\|\\ \leq \left[M\sum_{i=0}^{n-1}\left\|H^{-i}\right\|{ℒ}_{f}+Nn{ℒ}_{I_{k}}\right]\left\|\Theta -\Theta^{\prime }\right\|. \end{array}$$

This implies that *𝒜* is a contraction map using the Banach contraction principle, we say that system (1) has a unique solution.

## 4. *β*-Hyers–Ulam–Rassias Stability on Bounded Discrete Interval

To determine *β*-Hyers–Ulam–Rassias stability on the bounded discrete interval, we have one more assumption:

*G*_3_: there exist a constant *η*_*φ*_ > 0 and *φ*_*n*_ and a nondecreasing function *φ* ∈ *B*(*I*, *X*) such that

(19) $$\begin{array}{c} \sum_{i=0}^{n-1}\psi_{i}\leq \eta_{\phi }\phi_{n}. \end{array}$$

**Theorem 2 .**

System (1) is *β*-Hyers–Ulam–Rassias stable over discrete bounded interval, if *G*_1_, *G*_2_ and *G*_3_ are satisfied.

**Proof**

The solution of system (1) is as follows:

(20) $$\begin{array}{c} \Theta_{n}=H^{n}\Theta_{0}+H^{n-1}\sum_{i=0}^{n-1}H^{-i}B\zeta_{i}+H^{n-1}\sum_{i=0}^{n-1}H^{-i}f\left(i,\Theta_{i},\zeta_{i}\right)\\ +\sum_{n_{k}=0}^{n}T\left(n-n_{k}\right)I_{k}\left(n_{k},\Theta_{n_{k}-1},\zeta_{n_{k}-1}\right). \end{array}$$

Let *Y*_*n*_ be the solution of inequality (2), we have

(21) $$\begin{array}{c} \left\|Y_{n}-H^{n}\Theta_{0}-H^{n-1}\sum_{i=0}^{n}H^{-i}B\zeta_{i}-H^{n-1}\sum_{i=0}^{n}H^{-i}f\left(i,Y_{i},\zeta_{i}\right)-\sum_{n_{k}=0}^{n}T\left(n-n_{k}\right)I_{k}\left(n_{k},Y_{n_{k}-1},\zeta_{n_{k}-1}\right)\right\|\\ =\left\|H^{n}h_{n}+H^{n-1}\sum_{i=0}^{n-1}H^{-i}h_{i}+\overset{n}{\underset{n_{k}=0}{\sum }}T\left(n-n_{k}\right)h_{n_{k}}\right\|\leq M\varepsilon \Psi +M^{2}\text{ò}\sum_{i=0}^{n-1}\Psi_{i}+N\varepsilon \sum_{n_{k}=0}^{m}\Psi \\ =\left(M+N\right)\sum_{k=0}^{m}\text{ò}\Psi +\sum_{i=0}^{n-1}M^{2}\text{ò}\Psi_{i}\\ \leq \left(mM\text{ò}+M^{2}\varepsilon \eta_{\phi }\right)\left(\varphi_{n}+\varphi \right)\\ =M\text{ò}\left(m+M\eta_{\phi }\right)\left(\varphi_{n}+\varphi \right), \end{array}$$

thus, for each *n* ∈ {*n*_*k*_, *n*_*k*+1_,…}, we have

(22) $$\begin{array}{c} {\left\|Y_{n}-\theta_{n}\right\|}^{\beta }={\left\|\begin{array}{c} Y_{n}-H^{n}\Theta_{0}-H^{n-1}\sum_{i=0}^{n-1}H^{-i}B\zeta_{i}-H^{n-1}\sum_{i=0}^{n-1}H^{-i}f\left(i,\Theta_{i},\zeta_{i}\right)\\ -\sum_{n_{k}=0}^{n}T\left(n-n_{k}\right)I_{k}\left(n_{k},\Theta_{n_{k}-1},\zeta_{n_{k}-1}\right) \end{array}\right\|}^{\beta }\\ ={\left\|\begin{array}{c} Y_{n}-H^{n}\Theta_{0}-H^{n-1}\sum_{i=0}^{n-1}H^{-i}B\zeta_{i}-H^{n-1}\sum_{i=0}^{n-1}H^{-i}f\left(i,\Theta_{i},\zeta_{i}\right)\\ -\sum_{n_{k}=0}^{n}T\left(n-n_{k}\right)I_{k}\left(n_{k},\Theta_{n_{k}-1},\zeta_{n_{k}-1}\right)+H^{n-1}\sum_{i=0}^{n-1}H^{-i}f\left(i,Y_{i},\zeta_{i}\right)-H^{n-1}\\ \sum_{i=0}^{n-1}H^{-i}f\left(i,Y_{i},\zeta_{i}\right)+\sum_{n_{k}=0}^{n}T\left(n-n_{k}\right)I_{k}\left(n_{k},Y_{n_{k}-1},\zeta_{n_{k}-1}\right)\\ -\sum_{n_{k}=0}^{n}T\left(n-n_{k}\right)I_{k}\left(n_{k},Y_{n_{k}-1},\zeta_{n_{k}-1}\right) \end{array}\right\|}^{\beta }\\ \leq {\left(\left\|Y_{n}-H^{n}\Theta_{0}-H^{n-1}\sum_{i=0}^{n-1}H^{-i}B\zeta_{i}-H^{n-1}\sum_{i=0}^{n-1}H^{-i}f\left(i,Y_{i},\zeta_{i}\right)-\sum_{n_{k}=0}^{n}T\left(n-n_{k}\right)I_{k}\left(n_{k},Y_{n_{k}-1},\zeta_{n_{k}-1}\right)\right\|\right)}^{\beta }\\ +{\left(\left\|H^{n-1}\sum_{i=0}^{n-1}H^{-i}f\left(i,Y_{i},\zeta_{i}\right)-H^{n-1}\sum_{i=0}^{n-1}H^{-i}f\left(i,\Theta_{i},\zeta_{i}\right)\right\|\right)}^{\beta }\\ +{\left(\left\|\sum_{n_{k}=0}^{n}T\left(n-n_{k}\right)I_{k}\left(n_{k},Y_{n_{k}-1},\zeta_{n_{k}-1}\right)-\sum_{n_{k}=0}^{n}T\left(n-n_{k}\right)I_{k}\left(n_{k},\Theta_{n_{k}-1},\zeta_{n_{k}-1}\right)\right\|\right)}^{\beta }\\ \leq {\left(M\varepsilon \left(m+M\eta_{\phi }\right)\left(\varphi_{n}+\varphi \right)\right)}^{\beta }+{\left(M^{2}\sum_{i=0}^{n-1}{ℒ}_{f_{i}}\left\|Y_{i}-\Theta_{i}\right\|\right)}^{\beta }+{\left(N\sum_{k=1}^{m}{ℒ}_{I_{n_{k}}}\left\|Y_{n_{k}}-\Theta_{n_{k}}\right\|\right)}^{\beta }\\ \left\|Y_{n}-\Theta_{n}\right\|\leq 3^{\left(1/\beta \right)-1}\left[M\varepsilon \left(m+M\eta_{\phi }\right)\left(\varphi_{n}+\varphi \right)+M^{2}\sum_{i=0}^{n-1}{ℒ}_{f_{i}}\left\|Y_{i}-\Theta_{i}\right\|+N\sum_{k=1}^{m}{ℒ}_{I_{k}}\left\|Y_{n_{k}}-\Theta_{n_{k}}\right\|\right], \end{array}$$

by using the relation

(23) $$\begin{array}{c} {\left(x+y+z\right)}^{\gamma }\leq 3^{\gamma -1}\left(x^{\gamma }+y^{\gamma }+z^{\gamma }\right),\;\mathrm{where}\;k\;x,y,z\geq 0\;\mathrm{and}\;\gamma >1. \end{array}$$

Now, using Gronwall Lemma 2, we get

(24) $$\begin{array}{c} \left\|Y_{N}-\Theta_{n}\right\|\leq 3^{\left(1/\beta \right)-1}\left[M\varepsilon \left(m+M\eta_{\phi }\right)\left(\varphi_{n}+\varphi \right)\right]{\left(1+3^{\left(1/\beta \right)-1}N{ℒ}_{I_{k}}\right)}^{k}\exp\left(M^{2}3^{\left(1/\beta \right)-1}\sum_{i=0}^{n-1}{ℒ}_{f_{i}}\right). \end{array}$$

Now,

(25) $$\begin{array}{c} {\left\|Y_{n}-\Theta_{n}\right\|}^{\beta }\leq 3^{1-\beta }{\left[M\varepsilon \left(m+M\eta_{\phi }\right)\left(\varphi_{n}+\varphi \right)\right]}^{\beta }{\left(1+3^{\left(1/\beta \right)-1}N{ℒ}_{I_{k}}\right)}^{k\beta }\exp\left(M^{2}\beta 3^{\left(1/\beta \right)-1}\sum_{i=0}^{n-1}{ℒ}_{f_{i}}\right)\\ \leq 3^{1-\beta }{\left(M\varepsilon \left(m+M\eta_{\phi }\right)\right)}^{\beta }{\left(\varphi_{n}+\varphi \right)}^{\beta }{\left(1+3^{\left(1/\beta \right)-1}N{ℒ}_{I_{k}}\right)}^{k\beta }\exp\left(M^{2}\beta 3^{\left(1/\beta \right)-1}\sum_{i=0}^{n-1}{ℒ}_{f_{i}}\right)\\ \leq C_{f,N,M,\varphi ,\Psi }\varepsilon^{\beta }\left({\left(\varphi_{n}\right)}^{\beta }+\varphi^{\beta }\right), \end{array}$$

using (*x*+*y*)^*r*^ ≤ (*x*^*r*^+*y*^*r*^), *x*, *y* ≥ 0,  for any *r* ∈ {0,1}, where

(26) $$\begin{array}{c} C_{f,N,M,\varphi ,\Psi }=3^{1-\beta }{\left(M\varepsilon \left(m+M\eta_{\phi }\right)\right)}^{\beta }{\left(1+3^{\left(1/\beta \right)-1}N{ℒ}_{I_{k}}\right)}^{k\beta }\exp\left(M^{2}\beta 3^{\left(1/\beta \right)-1}\sum_{i=0}^{n-1}{ℒ}_{f_{i}}\right). \end{array}$$

Hence, system (1) is *β*-Hyers–Ulam–Rassaias stable. □

## 5. *β*-Hyers–Ulam–Rassias Stability on Unbounded Discrete Interval

To explain *β*-Hyers–Ulam–Rassias stability on an unbounded discrete interval, we must need the following assumptions:

*G*_4_: the operators family ‖*H*^*n*−*i*^‖ ≤ Me^*ω*(*n* − *i*)^ and ‖*T*(*n* − *n*_*k*_)‖ ≤ Me^*ω*(*n* − *n*_*k*_)^.

(27) $$\begin{array}{c} G_{5}:\sum_{i=0}^{n-1}{ℒ}_{f_{i}}\leq \kappa_{f}n+\varkappa_{f},\\ G_{6}:{ℒ}_{J}={\left(1+3^{\left(1/\beta \right)-1}M{ℒ}_{I_{n_{k}}}\right)}^{k}<\infty ,\\ G_{7}:\sum_{i=0}^{n-1}e^{\omega \left(n-i\right)+3^{1/\beta -1}M\kappa_{f}n}\Psi_{i}\leq \eta_{\varphi }\varphi_{n},\\ G_{8}:M_{1}=\sum_{n_{k}=0}^{m}e^{\omega \left(n-n_{k}\right)+3^{1/\beta -1}M\kappa_{f}n}. \end{array}$$

**Theorem 3 .**

Assume that *G*_1_ and *G*_3_ − *G*_8_ are holds, then system (1) is *β*-Hyers–Ulam–Rassias stable over a discrete unbounded interval.

**Proof**

The solution of system (1) is

(28) $$\begin{array}{c} \Theta_{n}=H^{n}\Theta_{0}+H^{n-1}\sum_{i=0}^{n-1}H^{-i}B\zeta_{i}+H^{n-1}\sum_{i=0}^{n-1}H^{-i}f\left(i,\Theta_{i},\zeta_{i}\right)\\ +\sum_{n_{k}=0}^{n}T\left(n-n_{k}\right)I_{k}\left(n_{k},\Theta_{n_{k}-1},\zeta_{n_{k}-1}\right). \end{array}$$

Let *Y*_*n*_ be the solution of inequality (2), we have

(29) $$\begin{array}{c} \left\|Y_{n}-H^{n}\Theta_{0}-H^{n-1}\sum_{i=0}^{n}H^{-i}B\zeta_{i}-H^{n-1}\sum_{i=0}^{n}H^{-i}f\left(i,Y_{i},\zeta_{i}\right)-\sum_{n_{k}=0}^{n}T\left(n-n_{k}\right)I_{k}\left(n_{k},Y_{n_{k}-1},\zeta_{n_{k}-1}\right)\right\|\\ =\left\|\sum_{i=0}^{n-1}H^{n-1-i}h_{i}+\sum_{n_{k}=0}^{n}T\left(n-n_{k}\right)h_{n_{k}}\right\|\leq \sum_{i=0}^{n-1}\left\|H^{n-1-i}\right\|{\left\|h\right\|}_{i}+\sum_{n_{k}=0}^{n}\left\|T\left(n-n_{k}\right)\right\|\left\|h_{n_{k}}\right\|\\ \leq M\left(\sum_{i=0}^{n-1}e^{\omega \left(n-i\right)}\varepsilon \Psi_{i}+\sum_{n_{k}=0}^{m}e^{\omega \left(n-n_{k}\right)}\varepsilon \Psi .\right. \end{array}$$

Now, for each *n* ∈ {*n*_*k*_, *n*_*k*+1_}, we have

(30) $$\begin{array}{c} {\left\|Y_{n}-\theta_{n}\right\|}^{\beta }={\left\|\begin{array}{c} Y_{n}-H^{n}\Theta_{0}-H^{n-1}\sum_{i=0}^{n-1}H^{-i}B\zeta_{i}-H^{n-1}\sum_{i=0}^{n-1}H^{-i}f\left(i,\Theta_{i},\zeta_{i}\right)\\ -\sum_{n_{k}=0}^{n}T\left(n-n_{k}\right)I_{k}\left(n_{k},\Theta_{n_{k}-1},\zeta_{n_{k}-1}\right) \end{array}\right\|}^{\beta }\\ ={\left\|\begin{array}{c} Y_{n}-H^{n}\Theta_{0}-H^{n-1}\sum_{i=0}^{n-1}H^{-i}B\zeta_{i}-H^{n-1}\sum_{i=0}^{n-1}H^{-i}f\left(i,\Theta_{i},\zeta_{i}\right)\\ -\sum_{n_{k}=0}^{n}T\left(n-n_{k}\right)I_{k}\left(n_{k},\Theta_{n_{k}-1},\zeta_{n_{k}-1}\right)+H^{n-1}\sum_{i=0}^{n-1}H^{-i}f\left(i,Y_{i},\zeta_{i}\right)\\ -H^{n-1}\sum_{i=0}^{n-1}H^{-i}f\left(i,Y_{i},\zeta_{i}\right)+\sum_{n_{k}=0}^{n}T\left(n-n_{k}\right)I_{k}\left(n_{k},Y_{n_{k}-1},\zeta_{n_{k}-1}\right)\\ -\sum_{n_{k}=0}^{n}T\left(n-n_{k}\right)I_{k}\left(n_{k},Y_{n_{k}-1},\zeta_{n_{k}-1}\right) \end{array}\right\|}^{\beta }\\ \leq {\left(\left\|\begin{array}{c} Y_{n}-H^{n}\Theta_{0}-H^{n-1}\sum_{i=0}^{n-1}H^{-i}B\zeta_{i}-H^{n-1}\sum_{i=0}^{n-1}H^{-i}f\left(i,Y_{i},\zeta_{i}\right)\\ -\sum_{n_{k}=0}^{n}T\left(n-n_{k}\right)I_{k}\left(n_{k},Y_{n_{k}-1},\zeta_{n_{k}-1}\right) \end{array}\right\|\right)}^{\beta }+\left(\left\|H^{n-1}\sum_{i=0}^{n-1}H^{-i}f\left(i,Y_{i},\zeta_{i}\right)\right\|\right)\\ -{\left.H^{n-1}\sum_{i=0}^{n-1}H^{-i}f\left(i,\Theta_{i},\zeta_{i}\right)\|\right)}^{\beta }+{\left(\left\|\begin{array}{c} \sum_{n_{k}=0}^{n}T\left(n-n_{k}\right)I_{k}\left(n_{k},Y_{n_{k}-1},\zeta_{n_{k}-1}\right)\\ -\sum_{n_{k}=0}^{n}T\left(n-n_{k}\right)I_{k}\left(n_{k},\Theta_{n_{k}-1},\zeta_{n_{k}-1}\right) \end{array}\right\|\right)}^{\beta }\\ \leq \left(M{\left(\sum_{i=0}^{n-1}e^{\omega \left(n-i\right)}\varepsilon \Psi_{i}+\sum_{n_{k}=0}^{m}e^{\omega \left(n-n_{k}\right)}\varepsilon \Psi \right)}^{\beta }+{\left(M\sum_{i=0}^{n-1}e^{\omega \left(n-i\right)}{ℒ}_{f_{i}}\left\|Y_{i}-\Theta_{i}\right\|\right)}^{\beta }\right.\\ +{\left(M\sum_{n_{k}=0}^{n}e^{\omega \left(n-n_{k}\right)}{ℒ}_{I_{n_{k}}}\left\|Y_{n_{k}-1}-\Theta_{n_{k}-1}\right\|\right)}^{\beta }, \end{array}$$

if we set ${\bar{Y}}_{n}=e^{-\omega n}Y_{n}$ and ${\bar{\Theta }}_{n}=e^{-\omega n}\Theta_{n}$, then we have

(31) $$\begin{array}{c} {\left\|{\bar{Y}}_{n}-{\bar{\Theta }}_{n}\right\|}^{\beta }\leq \left(M{\left(\sum_{i=0}^{n-1}e^{-\omega i}\varepsilon \Psi_{i}+\sum_{n_{k}=0}^{m}e^{-\omega n_{k}}\varepsilon \Psi \right)}^{\beta }+{\left(M\sum_{i=0}^{n-1}{ℒ}_{f_{i}}\left\|{\bar{Y}}_{i}-{\bar{\Theta }}_{i}\right\|\right)}^{\beta }\right.\\ +{\left(M\sum_{n_{k}=0}^{n}{ℒ}_{I_{n_{k}}}\left\|{\bar{Y}}_{n_{k}-1}-{\bar{\Theta }}_{n_{k}-1}\right\|\right)}^{\beta }, \end{array}$$

with the help of relation

(32) $$\begin{array}{c} {\left(x+y+z\right)}^{\gamma }\leq 3^{\gamma -1}\left(x^{\gamma }+y^{\gamma }+z^{\gamma }\right),\;\mathrm{where}\;x,y,z\geq 0\\ \gamma >1. \end{array}$$

we get

(33) $$\begin{array}{c} \left\|{\bar{Y}}_{n}-{\bar{\Theta }}_{n}\right\|\leq 3^{\left(1/\beta \right)-1}M\left(\sum_{i=0}^{n-1}e^{-\omega i}\varepsilon \Psi_{i}+\sum_{n_{k}=0}^{m}e^{-\omega n_{k}}\varepsilon \Psi \right)+3^{\left(1/\beta \right)-1}M\left(\sum_{i=0}^{n-1}{ℒ}_{f_{i}\left\|{\bar{Y}}_{i}-{\bar{\Theta }}_{i}\right\|}\right)\\ +3^{1/\beta -1}M\left(\sum_{n_{k}=0}^{n}{ℒ}_{I_{n_{k}}}\left\|{\bar{Y}}_{n_{k}-1}-{\bar{\Theta }}_{n_{k}-1}\right\|\right). \end{array}$$

Using Lemma 1, we have

(34) $$\begin{array}{c} \left\|{\bar{Y}}_{n}-{\bar{\Theta }}_{n}\right\|\leq 3^{\left(1/\beta \right)-1}M\varepsilon \left(\sum_{i=0}^{n-1}e^{-\omega i}\Psi_{i}+\sum_{n_{k}=0}^{m}e^{-\omega n_{k}}\Psi \right){\left(1+3^{\left(1/\beta \right)-1}M{ℒ}_{I_{n_{k}}}\right)}^{k}\exp\left(3^{\left(1/\beta \right)-1}M\sum_{i=0}^{n-1}{ℒ}_{f_{i}}\right). \end{array}$$

Resubmitting the values, we have

(35) $$\begin{array}{c} \left\|Y_{n}-\Theta_{n}\right\|\leq 3^{\left(1/\beta \right)-1}M\varepsilon \left(\sum_{i=0}^{n-1}e^{\omega \left(n-i\right)}\Psi_{i}+\sum_{n_{k}=0}^{m}e^{\omega \left(n-n_{k}\right)}\Psi \right){\left(1+3^{\left(1/\beta \right)-1}M{ℒ}_{I_{n_{k}}}\right)}^{k}\exp\left(3^{\left(1/\beta \right)-1}M\sum_{i=0}^{n-1}{ℒ}_{f_{i}}\right)\\ \leq 3^{1/\beta -1}M\varepsilon {ℒ}_{J}\left(\sum_{i=0}^{n-1}e^{\omega \left(n-i\right)}\Psi_{i}+\sum_{n_{k}=0}^{m}e^{\omega \left(n-n_{k}\right)}\Psi \right)\exp\left(3^{\left(1/\beta \right)-1}M\left(\kappa_{f}n+\varkappa_{f}\right)\right)\\ =3^{\left(1/\beta \right)-1}M\varepsilon {ℒ}_{J}\left(\sum_{i=0}^{n-1}e^{\omega \left(n-i\right)+3^{\left(1/\beta \right)-1}M\left(\kappa_{f}n+\varkappa_{f}\right)}\Psi_{i}+\sum_{n_{k}=0}^{m}e^{\omega \left(n-n_{k}\right)+3^{\left(1/\beta \right)-1}M\left(\kappa_{f}n+\varkappa_{f}\right)}\Psi \right)\\ =3^{\left(1/\beta \right)-1}M\varepsilon {ℒ}_{J}e^{3^{\left(1/\beta \right)-1M\varkappa_{f}}}\left(\sum_{i=0}^{n-1}e^{\omega \left(n-i\right)+3^{\left(1/\beta \right)-1}M\kappa_{f}n}\Psi_{i}+\sum_{n_{k}=0}^{m}e^{\omega \left(n-n_{k}\right)+3^{\left(1/\beta \right)-1}M\kappa_{f}n}\Psi \right)\\ \leq 3^{\left(1/\beta \right)-1}M\varepsilon {ℒ}_{J}e^{3^{1/\beta -1M\varkappa_{f}}}\left(\eta_{\varphi }\varphi +M_{1}\Psi \right)\\ \leq 3^{\left(1/\beta \right)-1}M\varepsilon {ℒ}_{J}e^{3^{\left(1/\beta \right)-1M\varkappa_{f}}}\left(M_{1}+\eta_{\varphi }\right)\left(\varphi_{n}+\Psi \right)\\ {\left\|Y_{n}-\Theta_{n}\right\|}^{\beta }\leq {ℋ}_{M,{ℒ}_{J},\eta_{\varphi },M_{1}}\varepsilon^{\beta }\left(\varphi_{n}^{\beta }+\Psi^{\beta }\right), \end{array}$$

where

(36) $$\begin{array}{c} {ℋ}_{M,{ℒ}_{J},\eta_{\varphi },M_{1}}=3^{1-\beta }{\left(M{ℒ}_{J}e^{3^{\left(1/\beta \right)-1M\varkappa_{f}}}\left(M_{1}+\eta_{\varphi }\right)\right)}^{\beta }>0. \end{array}$$

Thus, system (1) is *β*-Hyers–Ulam–Rassias stable.

**Remark 3 .**

Wang et al. [18] studied the *β*-Hyers–Ulam stability and *β*-Hyers–Ulam–Rassias stability for a system of impulsive differential equations as we know that difference equations relate to differential equations as discrete mathematics relate to continuous mathematics. The system of impulsive difference equations used in this article is analogous to the system of impulsive differential equations used in [18]. Thus, the findings of this article are the discrete version of the work of Wang et al. [18].

## 6. Generalized *β*-Hyers–Ulam–Rassias Stability

In this section, we present the generalized *β*-Hyers–Ulam–Rassias stability, for which we need the following assumptions:

(37) $$\begin{array}{c} G_{9}:3^{\left(1/\beta \right)-1}M\varepsilon \prod_{n_{k}=0}^{n}\left(1+3^{\left(1/\beta \right)-1}M{ℒ}_{I_{n_{k}}}\right)\left(\sum_{i=0}^{n-1}e^{\omega \left(n-i\right)+3^{\left(1/\beta \right)-1}M\sum_{i=0}^{n-1}{ℒ}_{f_{i}}}\Psi_{i}\right)\leq \eta_{\varphi }\varphi_{n},\\ G_{10}:3^{\left(1/\beta \right)-1}M\varepsilon \prod_{n_{k}=0}^{n}\left(1+3^{\left(1/\beta \right)-1}M{ℒ}_{I_{n_{k}}}\right)\left(\sum_{n_{k}=0}^{m}e^{\omega \left(n-n_{k}\right)+3^{\left(1/\beta \right)-1}M\sum_{i=0}^{n-1}{ℒ}_{f_{i}}}\Psi \right)\leq \eta_{\Psi }\Psi_{k+1}. \end{array}$$

**Theorem 4 .**

Assume that *G*_1_ and *G*_3_ − *G*_10_ are satisfied, then system (1) is generalized *β*-Hyers–Ulam–Rassias stable.

**Proof**

The solution of system (1) is as follows:

(38) $$\begin{array}{c} \Theta_{n}=H^{n}\Theta_{0}+H^{n-1}\sum_{i=0}^{n-1}H^{-i}B\zeta_{i}+H^{n-1}\sum_{i=0}^{n-1}H^{-i}f\left(i,\Theta_{i},\zeta_{i}\right)\\ +\sum_{n_{k}=0}^{n}T\left(n-n_{k}\right)I_{k}\left(n_{k},\Theta_{n_{k}-1},\zeta_{n_{k}-1}\right). \end{array}$$

Let *Y*_*n*_ be the solution of inequality (2), we have

(39) $$\begin{array}{c} \left\|Y_{n}-H^{n}\Theta_{0}-H^{n-1}\sum_{i=0}^{n}H^{-i}B\zeta_{i}-H^{n-1}\sum_{i=0}^{n}H^{-i}f\left(i,Y_{i},\zeta_{i}\right)-\sum_{n_{k}=0}^{n}T\left(n-n_{k}\right)I_{k}\left(n_{k},Y_{n_{k}-1},\zeta_{n_{k}-1}\right)\right\|\\ =\left\|\sum_{i=0}^{n-1}H^{n-1-i}h_{i}+\sum_{n_{k}=0}^{n}T\left(n-n_{k}\right)h_{n_{k}}\right\|\leq \sum_{i=0}^{n-1}\left\|H^{n-1-i}\right\|\left\|h_{i}\right\|+\sum_{n_{k}=0}^{n}\left\|T\left(n-n_{k}\right)\right\|\left\|h_{n_{k}}\right\|\\ \leq M\left(\sum_{i=0}^{n-1}e^{\omega \left(n-i\right)}\varepsilon \Psi_{i}+\sum_{n_{k}=0}^{m}e^{\omega \left(n-n_{k}\right)}\varepsilon \Psi \right). \end{array}$$

Now, for each *n* ∈ {*n*_*k*_, *n*_*k*+1_}, we have

(40) $$\begin{array}{c} {\left\|Y_{n}-\theta_{n}\right\|}^{\beta }={\left\|\begin{array}{c} Y_{n}-H^{n}\Theta_{0}-H^{n-1}\sum_{i=0}^{n-1}H^{-i}B\zeta_{i}-H^{n-1}\sum_{i=0}^{n-1}H^{-i}f\left(i,\Theta_{i},\zeta_{i}\right)\\ -\sum_{n_{k}=0}^{n}T\left(n-n_{k}\right)I_{k}\left(n_{k},\Theta_{n_{k}-1},\zeta_{n_{k}-1}\right) \end{array}\right\|}^{\beta }\\ ={\left\|\begin{array}{c} Y_{n}-H^{n}\Theta_{0}-H^{n-1}\sum_{i=0}^{n-1}H^{-i}B\zeta_{i}-H^{n-1}\sum_{i=0}^{n-1}H^{-i}f\left(i,\Theta_{i},\zeta_{i}\right)\\ -\sum_{n_{k}=0}^{n}T\left(n-n_{k}\right)I_{k}\left(n_{k},\Theta_{n_{k}-1},\zeta_{n_{k}-1}\right)+H^{n-1}\sum_{i=0}^{n-1}H^{-i}f\left(i,Y_{i},\zeta_{i}\right)\\ -H^{n-1}\sum_{i=0}^{n-1}H^{-i}f\left(i,Y_{i},\zeta_{i}\right)+\sum_{n_{k}=0}^{n}T\left(n-n_{k}\right)I_{k}\left(n_{k},Y_{n_{k}-1},\zeta_{n_{k}-1}\right)\\ -\sum_{n_{k}=0}^{n}T\left(n-n_{k}\right)I_{k}\left(n_{k},Y_{n_{k}-1},\zeta_{n_{k}-1}\right) \end{array}\right\|}^{\beta }\\ \leq {\left(\left\|Y_{n}-H^{n}\Theta_{0}-H^{n-1}\sum_{i=0}^{n-1}H^{-i}B\zeta_{i}-H^{n-1}\sum_{i=0}^{n-1}H^{-i}f\left(i,Y_{i},\zeta_{i}\right)-\sum_{n_{k}=0}^{n}T\left(n-n_{k}\right)I_{k}\left(n_{k},Y_{n_{k}-1},\zeta_{n_{k}-1}\right)\right\|\right)}^{\beta }\\ +{\left(\left\|H^{n-1}\sum_{i=0}^{n-1}H^{-i}f\left(i,Y_{i},\zeta_{i}\right)-H^{n-1}\sum_{i=0}^{n-1}H^{-i}f\left(i,\Theta_{i},\zeta_{i}\right)\right\|\right)}^{\beta }\\ +{\left(\left\|\sum_{n_{k}=0}^{n}T\left(n-n_{k}\right)I_{k}\left(n_{k},Y_{n_{k}-1},\zeta_{n_{k}-1}\right)-\sum_{n_{k}=0}^{n}T\left(n-n_{k}\right)I_{k}\left(n_{k},\Theta_{n_{k}-1},\zeta_{n_{k}-1}\right)\right\|\right)}^{\beta }\\ \leq \left(M{\left(\sum_{i=0}^{n-1}e^{\omega \left(n-i\right)}\varepsilon \Psi_{i}+\sum_{n_{k}=0}^{m}e^{\omega \left(n-n_{k}\right)}\varepsilon \Psi \right)}^{\beta }+{\left(M\sum_{i=0}^{n-1}e^{\omega \left(n-i\right)}{ℒ}_{f_{i}}\left\|Y_{i}-\Theta_{i}\right\|\right)}^{\beta }\right.\\ +{\left(M\sum_{n_{k}=0}^{n}e^{\omega \left(n-n_{k}\right)}{ℒ}_{I_{n_{k}}}\left\|Y_{n_{k}-1}-\Theta_{n_{k}-1}\right\|\right)}^{\beta }, \end{array}$$

if we set ${\bar{Y}}_{n}=e^{-\omega n}Y_{n}$ and ${\bar{\Theta }}_{n}=e^{-\omega n}\Theta_{n}$, then we have

(41) $$\begin{array}{c} {\left\|{\bar{Y}}_{n}-{\bar{\Theta }}_{n}\right\|}^{\beta }\leq \left(M{\left(\sum_{i=0}^{n-1}e^{-\omega i}\varepsilon \Psi_{i}+\sum_{n_{k}=0}^{m}e^{-\omega n_{k}}\varepsilon \Psi \right)}^{\beta }+{\left(M\sum_{i=0}^{n-1}{ℒ}_{f_{i}}\left\|{\bar{Y}}_{i}-{\bar{\Theta }}_{i}\right\|\right)}^{\beta }\right.\\ +{\left(M\sum_{n_{k}=0}^{n}{ℒ}_{I_{n_{k}}}\left\|{\bar{Y}}_{n_{k}-1}-{\bar{\Theta }}_{n_{k}-1}\right\|\right)}^{\beta }, \end{array}$$

with the help of relation

(42) $$\begin{array}{c} {\left(x+y+z\right)}^{\gamma }\leq 3^{\gamma -1}\left(x^{\gamma }+y^{\gamma }+z^{\gamma }\right),\;\mathrm{where}\;x,y,z\geq 0\\ \gamma >1, \end{array}$$

we get

(43) $$\begin{array}{c} \left\|{\bar{Y}}_{n}-{\bar{\Theta }}_{n}\right\|\leq 3^{\left(1/\beta \right)-1}M\left(\sum_{i=0}^{n-1}e^{-\omega i}\varepsilon \Psi_{i}+\sum_{n_{k}=0}^{m}e^{-\omega n_{k}}\varepsilon \Psi \right)+3^{\left(1/\beta \right)-1}M\left(\sum_{i=0}^{n-1}{ℒ}_{f_{i}}\left\|{\bar{Y}}_{i}-{\bar{\Theta }}_{i}\right\|\right)\\ +3^{\left(1/\beta \right)-1}M\left(\sum_{n_{k}=0}^{n}{ℒ}_{I_{n_{k}}}\left\|{\bar{Y}}_{n_{k}-1}-{\bar{\Theta }}_{n_{k}-1}\right\|\right), \end{array}$$

using, we have

(44) $$\begin{array}{c} \left\|{\bar{Y}}_{n}-{\bar{\Theta }}_{n}\right\|\leq 3^{\left(1/\beta \right)-1}M\varepsilon \prod_{n_{k}=0}^{n}\left(\sum_{i=0}^{n-1}e^{-\omega i}\Psi_{i}+\sum_{n_{k}=0}^{m}e^{-\omega n_{k}}\Psi \right)\left(1+3^{\left(1/\beta \right)-1}M{ℒ}_{I_{n_{k}}}\right)\exp\left(3^{\left(1/\beta \right)-1}M\sum_{i=0}^{n-1}{ℒ}_{f_{i}}\right), \end{array}$$

resubmitting the values, we have

(45) $$\begin{array}{c} \left\|Y_{n}-\Theta_{n}\right\|\leq 3^{\left(1/\beta \right)-1}M\varepsilon \prod_{n_{k}=0}^{n}\left(\sum_{i=0}^{n-1}e^{\omega \left(n-i\right)}\Psi_{i}+\sum_{n_{k}=0}^{m}e^{\omega \left(n-n_{k}\right)}\Psi \right)\left(1+3^{\left(1/\beta \right)-1}M{ℒ}_{I_{n_{k}}}\right)\exp\left(3^{\left(1/\beta \right)-1}M\sum_{i=0}^{n-1}{ℒ}_{f_{i}}\right)\\ =3^{\left(1/\beta \right)-1}M\varepsilon \prod_{n_{k}=0}^{n}\left(\begin{array}{c} \sum_{i=0}^{n-1}e^{\omega \left(n-i\right)+3^{\left(1/\beta \right)-1}M\sum_{i=0}^{n-1}{ℒ}_{f_{i}}}\Psi_{i}\\ +\sum_{n_{k}=0}^{m}e^{\omega \left(n-n_{k}\right)+3^{\left(1/\beta \right)-1}M\sum_{i=0}^{n-1}{ℒ}_{f_{i}}}\Psi \end{array}\right)\left(1+3^{\left(1/\beta \right)-1}M{ℒ}_{I_{n_{k}}}\right), \end{array}$$

At last, we obtain

(46) $$\begin{array}{c} \left\|Y_{n}-\Theta_{n}\right\|\leq 3^{1/\beta -1}M\varepsilon \left(\eta_{\varphi }\varphi_{n}+\eta_{\Psi }\Psi_{k+1}\right)\\ \leq 3^{1/\beta -1}M\varepsilon \left(\eta_{\varphi }+\eta_{\Psi }\right)\left(\varphi_{n}+\Psi_{k+1}\right)\\ {\left\|Y_{n}-\Theta_{n}\right\|}^{\beta }=3^{1-\beta }M^{\beta }\varepsilon^{\beta }{\left(\eta_{\varphi }+\eta_{\Psi }\right)}^{\beta }{\left(\varphi_{n}+\Psi_{k+1}\right)}^{\beta }\\ =J_{M,\eta_{\Psi },\eta_{\phi },f}\varepsilon^{\beta }\left(\varphi_{n}^{\beta }+\Psi_{k+1}^{\beta }\right), \end{array}$$

where

(47) $$\begin{array}{c} J_{M,\eta_{\Psi },\eta_{\phi },f}=3^{1-\beta }M^{\beta }{\left(\eta_{\varphi }+\eta_{\Psi }\right)}^{\beta }>0. \end{array}$$

Hence, system (1) is generalized *β*-Hyers–Ulam–Rassias stable.

## 7. Example

The impulsive difference system is as follows:

(48) $$\begin{array}{c} \left\{\begin{array}{c} \Theta_{n+1}=H\Theta_{n}+B\zeta_{n}+f\left(n,\Theta_{n},\zeta_{n}\right),\;n\in \left\{0,1,2,3\right\},\\ \Theta_{0},\;n=0,\\ \Delta \left(\Theta_{n_{k}}\right)=I_{k}\left(n,\Theta_{n_{k}-1},\zeta_{n_{k}-1}\right)=\frac{1}{3r^{2}}\left(\Theta_{n_{k}-1}\right),\;k=1,2,3,\ldots ,m. \end{array}\right. \end{array}$$

Assumption *G*_1_ and *G*_2_ are holds if *N*=1 and *M*=1. Clearly, assumptions *G*_5_ and *G*_6_ are hold if *κ*_*f*_=0 and

(49) $$\begin{array}{c} {ℒ}_{J}=\text{sup}\prod_{r=1}^{\infty }\left(1+3^{\left(1/\beta \right)}M{ℒ}_{I_{n_{k}}}\right)\\ =\text{sup}\prod_{r=1}^{\infty }\left(1+\frac{1}{r^{2}}\right)\leq e^{\sum_{l=1}^{\infty }\left(1/r^{2}\right)}\leq e^{\left(\pi^{2}/6\right)}. \end{array}$$

Also, ù_*f*_=0. Now, set *φ*_*n*_=*e*^*n*^ and *ψ*=1, then *G*_7_ holds if *η*_*ϕ*_=1/2. *G*_8_ holds if *e*^2^/*e*^2^ − 1. Thus, system (3) is 1/2− Hyers–Ulam–Rassias stable with respect to $\left(\sqrt{e^{n}},1\right)$ on *Z*_+_ and ${ℋ}_{M,{ℒ}_{𝒥},\eta_{\varphi },M_{1}}=\sqrt{3}e^{\pi^{2}/r^{2}}{\left(1/2+e^{2}/e^{2}-1\right)}^{1/2}$.

## 8. Conclusion

Nowadays, studies on the qualitative behavior of impulsive difference equations have a significant contribution to the literature. In particular, the discussion regarding the *β*-Hyers–Ulam–Rassias stability of difference equations has been considered as one of the important topics of the literature, in which different types of conditions have been used in the form of inequalities, and most results have been obtained through discrete Gronwall inequality. In this paper, we have investigated the existence and uniqueness of the solution through the Banach contraction principle and *β*-Hyers–Ulam–Rassias stability of the impulsive difference system with the help of Gronwall inequality.

## Data Availability

The data used to support the findings of this study are available from the corresponding author upon request.
